# Forensic Medicine in South Africa: Associations between Medical Practice and Legal Case Progression and Outcomes in Female Murders

**DOI:** 10.1371/journal.pone.0028620

**Published:** 2011-12-14

**Authors:** Naeemah Abrahams, Rachel Jewkes, Lorna J. Martin, Shanaaz Mathews

**Affiliations:** 1 Gender and Health Research Unit, South African Medical Research Council, Cape Town, South Africa; 2 Gender and Health Research Unit, South African Medical Research Council, Gauteng, South Africa; 3 Forensic Medicine and Toxicology, Faculty of Health Sciences, University of Cape Town, Cape Town, South Africa; Vanderbilt University, United States of America

## Abstract

**Background:**

Forensic medicine has been largely by-passed by the tide of health systems research and evidence based medicine. Murder victims form a central part of forensic medical examiners' case load, and women murdered by intimate partners are an important subgroup, representing the most severe form and consequence of intimate partner violence. Our aim was to describe the epidemiology of female murder in South Africa (by intimate and non-intimate partners); and to describe and compare autopsy findings, forensic medical management of cases and the contribution of these to legal outcomes.

**Methods:**

We did a retrospective national study in a proportionate random sample of 25 medico-legal laboratories to identify all homicides in 1999 of women aged 14 years and over. Data were abstracted from the mortuary file and autopsy report, and collected from a police interview.

**Findings:**

In 21.5% of cases the perpetrator was convicted. Factors associated with a conviction for the female murders included having a history of intimate partner violence 1.18 (95%CI: 0.16–2.20), weapon recovered 1.36 (95% CI:0.58–2.15) and a detective visiting the crime scene 1.57 (95% CI:0.14–3.00). None of the forensic medical activities increased the likelihood of a conviction.

**Conclusion:**

The findings raise important questions about the role of forensic medicine in these cases.

## Introduction

In South Africa murder is routine. The country has one of the highest homicide and assault rates in the world [1], [2] and a consequence is that for those practicing clinical forensic medicine, as well as police and courts personnel, violent crime is the bread and butter of their daily work. Forensic medicine is an intersection between medicine and the law which embraces a range of activities conducted to build an effective prosecution to assist courts in reaching the correct decision [3], [4]. It can facilitate and influence legal cases through what is done and how well it is documented, and how robustly the findings and conclusions are presented in court. In homicide cases a large range of other factors influence the progression of cases in the legal system and case outcomes, most notably how well the cases are investigated and prepared for court by the police and prosecutors, including the extent to which there is evidence to link the alleged perpetrator to the crime. These in turn are influenced by a wide range of aspects of the broader environment including levels of resources, but also the personnel involved, notably their skills, experience, commitment and attitudes towards the cases.

Over the last few years South Africa has implemented a range of measures to extend and improve the practice of forensic pathology, through reorganizing management and introducing new training initiatives [5], [6]. Yet both locally and internationally, there has been remarkably little research into the way in which the specialty's practices influence the criminal prosecution that it seeks to assist and which aspects of these are of relatively greater importance. A review of research on the role of the medical legal specialty was done for sexual assault and showed the limited role of clinical forensic medicine to the legal outcome of such cases [7]. A recent South African study found that good documentation of injuries have been found to be associated with the commencement and convictions of rape trials [8]. These are encouraging but similar studies on the role of forensic pathology in homicide have not been done. These questions form a critical part of the evidence base that could influence the direction of the specialty particularly in a setting that is generally resource constrained and where homicides are common.

As part of a national study data was collected on the background of cases, police investigations and outcomes and forensic medical management. This dataset provides an opportunity to investigate some of the factors associated with legal processes i.e. whether someone was charged for the murder and whether a conviction was obtained. We are aware that a range of factors could potentially influence the legal outcome of female homicides but this study allows us to explore, describe and test hypothesis about which forensic factors are associated with the outcome.

## Methods

### Ethics Statement

The study protocol was approved by the Ethics Committee of the South African Medical Research Council. Consent procedures were followed for access to the data at the mortuaries and police. Additionally, interviews with the police were only done after written informed consent was given.

This analysis is based on a national retrospective mortuary-based (medical legal laboratories) study. Detailed methods and main findings have been reported elsewhere [9]–[12]. In brief all mortuaries operating in South Africa in 1999 formed the sampling frame and was stratified by size based on the number of autopsies performed per annum (small <500 autopsies, medium 500–1499 autopsies, large >1499 autopsies). A stratified random sample of 25 mortuaries was drawn using proportional allocation and all women aged 14 and above, who had been killed between 1 January–31 December 1999 and whose bodies were taken to the sampled mortuaries were identified using the death registers.

All gunshots, head injuries, poisonings, hangings, decomposed bodies or any cases where the cause of death was ‘undetermined’ or ‘unknown’ were initially included. Cases were finally classified as homicide or non-homicide after review of the autopsy report and data collected from police. The police data collection was either through telephonic or face-to-face interview with the case's primary investigating officer (in 53.7% of cases), the police station's commanding officer (27.1%), or direct inspection of the police docket by a researcher (19.2%). In order to check the accuracy of the recording of legal case outcome in the police dockets, we validated a sub-sample of reports of the legal outcome of cases against court records and found a 100% convergence.

The information recorded from the mortuary data included case identification, details of death and social and demographic characteristics of victims. The autopsy reports provided information on cause of death, forensic pathology information, specimen collection, photography and number and description of injuries. Data from the police included: social and demographic characteristics of the suspect information about the case investigation and outcome. Following convention in this field of research and surveillance established by the Supplementary Homicide Reports of the Bureau of Justice [13], the suspect of the homicide was defined as the person the investigating officer perceived to be primarily responsible for the murder, whether or not he was charged with a murder.

### Outcome and other variables

Two outcome variables are of interest in this analysis: whether someone was charged and if charged whether someone was convicted. The charged group included all cases that proceeded to the court process irrespective of the type of charge (could have been lesser charges such as culpable homicide) while the non-charged group included those cases where the suspects remained unknown or were known and strongly suspected of committing the murder but never arrested. The convicted group were those where a perpetrator was convicted for homicide or culpable homicide, while the non-convicted group were those that were charged but the persons were acquitted because of lack of evidence. The conviction group excluded cases that were still on trial, those suspects but found to be psychologically unfit to stand trial, those cases with an outcome of accidental death or due to self-defence and cases where the cause of death remained undetermined.

The aspects of forensic medical management of interest were: the extent of the autopsy (full or partial); whether the crime scene was visited by the medical examiner; whether specimens were collected to be examined in a forensic science laboratory for DNA, alcohol, toxicology or other investigations, including nail scrapings, head hair, blood, clothing, histology and genital swabs; whether photos were taken to document forensic findings; and the quality of the autopsy report. A score was developed to assess the quality of a post mortem report and this was based on the international standards for writing of autopsy reports [14] and guidelines developed by the South African National Department of Health for the Performance of Post mortems (known as the GW 7/71). These standards require descriptions for three external examinations: description of the location of the lesions; pathological descriptions and description of wound dimensions. A five point score was used to determine the proportion of correct descriptions for each of the three dimensions (a scale of 0–5 for each of the three dimensions giving a maximum quality score of 15). For example a score of five indicated that 100% of the external examination were described correctly while a score of 1 indicated 0–25% of the external examinations were described correctly. An adequate report had to have all three aspects scored five while an inadequate report did not meet one or more of the adequate criteria. A superior report had all the adequate criteria in addition to one or more further identifying features such as elaboration of injuries; measurement of organs or description of anatomy and pathology found during dissection. This assessment was done by the forensic pathologist on the research team (LJM) during the review of the reports.

We had variables related to other aspects of the investigation, notably whether a weapon was found; whether the investigating officer went to the crime scene; the taking of crime photos; whether victim- suspect relationship was classified as ‘intimate’ (included a current or ex- husband or boyfriend, other sexual partner [including lesbian] or rejected suitor) and whether the suspects was known to have been previously violent (history of intimate partner violence).

We analyzed the weighted data using Stata Version 11.0 [15]. The distribution of variables related to the forensic medical management of cases, case investigations and victim and suspect/perpetrator characteristics are described by outcome variable using frequencies, proportions and means (with 95% confidence intervals). For categorical variables the associations are presented with odds ratios and 95% confidence intervals using the Chi-square test. We fitted a multi-state statistical model to examine the transition from the state of charged to the state of convicted. In the absence of duration in each state, we set this modelling approach within competing risk model where the perpetrator could move to three states: “not charged”, “charged and not convicted” and “charged and convicted”. We fitted these using a multinomial logistic regression by using two contrasts charged and not convicted vs. not charged and charged and not convicted vs. charged and convicted. We adjusted this analysis for race, urban rural status, age and regional location of murder (provinces) to control for possible confounding. We considered the demographic, investigative and medical forensic variables in the building of the models using a backward stepwise process.

## Results

**Figure 1:**
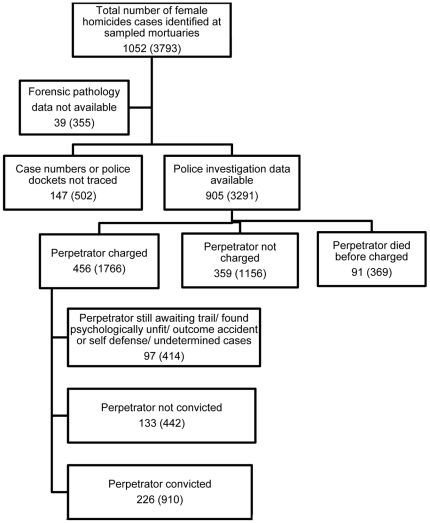
Flow diagram of the charged and convicted female homicide cases (weighted figures in brackets).

All sampled mortuaries contributed data to the study. Figure 1 presents a flow diagram showing the total sample of murdered women and the sub-groups for which complete information was available for the analysis.

Table 1 presents the data on the forensic pathology services for all the homicides and less than a quarter (20.3%) of the post mortems were done at academic institutions and two thirds were performed by medical practitioners with no formal forensic pathology training (66.2%) while two-fifths of the post mortem reports (40%) was found to be below the acceptable standard. Criminal charges were processed for 43.3% (95% CI: 40.1%–46.3%) (456/1052) of the female murders and a conviction of a perpetrator secured for 21.5% (95% CI: 18.9%–23.9%) (226/1052). A conviction was achieved for nearly half of those charged (49.6% 226/456).

**Table 1:** Frequency distribution of forensic pathology services: all cases (weighted data).

	% (95% CI)
**Post mortem done at academic centre**	
Yes	20.3 (6.6–48.1)
No	79.7 (51.9–93.4)
**Medical practitioner qualification**	
Specialist training	17.2 (6.2–39.4)
Some training	16.6 (6.8–35.3)
No training	66.2 (43.7–83.1)
**Standard of post mortem report**	
Superior	25.8 (11.6–48.1)
Adequate	35.2 (21.6–51.6)
Below adequate	40.0 (24.5–55.6)

Table S1 presents the comparisons of the socio demographics profile of victims and the suspects and case investigation characteristics for those charged and those convicted. A decreased likelihood of being charged was associated with older victims while an increased likelihood of being charged was associated with victims of the colored race group compared to victims from the African race(OR:2.85;95% CI:1.52–5.35); suspects being intimate partners (OR: 2.77 95% CI: 1.48–9.48); the women being killed in her home (OR:1.90:95% CI:1.07–3.40) and a weapon being found during the investigation (OR:16.02; 95% CI:8.66–29.64).

In contrast conviction was not associated with the age of victims but was more likely if the victim was of coloured race (OR:2.83; 95% CI: 1.12–7.11) and the suspect as well (OR: 3.75 95% CI: 1.21–11.61). As for being charged a history of intimate partner violence was associated with a greater likelihood of a conviction (OR:2.23; 95%CI:1.01–4.89). Conviction was also more likely if the crime scene was visited by the investigating officer (OR: 5.00; 95%CI: 1.78–14.04) and if a weapon was found by the police.

Table S2 compares variables related to the forensic management of the cases and injuries with the two outcomes of interest. In general specimen collection for evidence was low and only few forensic variables were associated with a suspect either charged or being convicted. Suspects were less likely to be charged if specimens were collected for DNA analysis (OR: 0.44; 95%CI: 0.26–0.72), if genital swab specimens were collected (OR: 0.41; 95%CI: 0.22–0.76) and if evidence of rape was reported in the report (OR:0.40; 95%CI: 0.18–0.87). None of the autopsy practices were associated with a conviction except the collection of specimens from nail scrapping that was positively associated with conviction of a perpetrator (OR: 4.30; 95%Ci: 1.36–13,53).

Table 2 shows the multinomial logistic regression model for the regression of the three states: charged and not convicted (reference), not charged and charged and convicted. Finding a weapon was significantly associated with both the state of not charged and the state of convicted compared to the reference group but in different directions with a decrease likelihood for those not charged (*p* = <0.001) and an increase likelihood for those convicted (*p* = 0.002). An increase likelihood of a conviction was also associated with the case having a history of intimate partner violence (*p* = 0.025) and the investigator visiting the crime scene (*p* = 0.033) compared to charged but not convicted. These associations were not found for the not charged state. In addition none of the forensic pathology variables increased the likelihood of any of the outcomes.

**Table 2:** Multinomial logistic regression showing the associations between the state of charged and not convicted with not charged and charged and convicted (reference group is charged and not convicted).

	Not Charged			Charged and convicted		
	Coefficient	95% CI	*p* –Value	Coefficient	95% CI	*p* –Value
Weapon found	−1.73	−2.34–1.13	<0.001	1.36	0.58–2.15	0.002
Investigation officer visited the crime scene	−.99	−2.52–0.53	0.192	1.57	0.14–3.00	0.033
History of intimate partner violence	1.10	−2.96–.75	0.231	1.18	0.16–2.20	0.025

Adjusted for age, urban/rural, race and regional location of murder [provinces].

## Discussion

The female homicide study provided us with an opportunity to explore the role of medical forensic examination on the legal outcome of homicide cases. Few associations were found between conviction and any measure that may have indicated a higher quality autopsy or crime scene investigation. Samples that could be examined for DNA were taken in 17.2% of all cases and 13.4% among the convicted cases. Much attention has been given to the potential powerful role of DNA analysis in solving crimes [16], [17] but DNA evidence isn't always available and depending on the victim/alleged perpetrator relationship, does not always assist the courts. In South Africa all specimens are stored and currently only analysed when request is made from a prosecutor.

However many factors are involved in a conviction, stretching from the crime scene to court, and the post mortem report may therefore not play a decisive role in many cases. These other factors include apprehension of the suspect, his/her plea, and the quality of the investigation and prosecution- the latter being the central theme of a recent book depicting the investigation and collection of evidence of a high profile murder of a young South Africa woman [18].

We controlled for urban rural differences in the analysis since the majority of the cases in this study were examined by non-specialists, indicating that they came from secondary level hospitals or rural centres where the police investigation may have been different from those in larger cities because of better cooperation from communities and the greater likelihood that the person sought was known. It is also possible that relatively less sophisticated suspects from rural areas may have been more likely to plead guilty when in court or had inferior defence lawyers. Future studies should attempt to explore these differences better.

It is encouraging however that our study showed that a visit to the crime scene by an investigating officer, a basic investigative activity, contributed to a conviction. Similarly the finding of a weapon was also associated with a conviction. It is even more encouraging to find that a history of intimate partner violence contributes to a conviction. This clearly shows that prosecutors and courts are considering such violence history as having evidentiary value and it also indicates that the police are collecting this data as part of their investigation. Collecting such data should become part of the routine investigation for crimes against all women – in particular murders and assaults as the larger study from which the data was drawn has shown that half of the women murdered in South Africa are killed by a intimate partner [10].

This study has provided some insight into the practices of forensic medical examiners in South Africa. There are no comparable data available, but this study has shown that in almost all cases it was stated that a full (usually) or partial autopsy was performed. Forensic medical examiners rarely visited crime scenes, and usually did not collect specimens or materials that could have traces of DNA, or be used for any other laboratory investigations (apart from blood alcohol). The negative association between being charged and some of the specimen collected could have been due to chance since these associations did not remain in the adjusted analysis. We have shown that neither the extensiveness of the autopsy, nor reports of high standards, nor collection of specimens, positively contributed towards a conviction of a suspect. Although this is most likely in our opinion to be due to unmeasured confounders it may also be an indication of a mismatch between the quality of forensic medicine practice and the quality of police investigations and operations in court. Another explanation could be that pathology reports are often submitted as evidence but are not interrogated and it is possible that without this they may not influence the outcome of cases. The experience of the forensic pathologist on the research team has been that they are seldom called to court and only if the autopsy report is in dispute.

There has been little research internationally on the role of forensic evidence on legal outcomes of cases. In this respect our study findings are similar to those of studies on the role of medico-legal evidence in the outcomes of rape cases. A recent review of this evidence established that it seldom contributes positively to case outcomes [7]. Our study found that convictions were more likely in cases where an investigator visited the crime scene, if a weapon was found and if a history of intimate partner violence was known by the investigator. These findings are encouraging and show that basic police investigations remain the key factors in the seeking of justice for these murders.

Our study has some limitations, the most important of which is that we depended on police data. Inevitably data were missing for cases where the police had few leads. As a result we have a considerable amount of missing data on suspects. There is also information which we would have liked but was unavailable due to poor management of cases and information systems. Inevitably we could only collect variables that were available in the police dockets. Lack of adequate crime data is not unique to South Africa. In North America the supplemental homicide report was implemented to overcome similar limitations, but they remain problematic [13].

These findings raise very important questions about the role of forensic medicine in South Africa and suggest the need for research in other settings. Whilst the study findings may not be generalisable to other countries, they raise important issues for debate and further research within the specialty. Specialists in the field both locally and internationally have raised concerns about the lack of a scientific approach to the setting of standards for autopsies, their reports and lack of audit [6], [19]–[21]. Where the murder rate is high, undertaking full autopsies is very resource intensive, as well as psychologically stressful, for forensic medical examiners. There is clearly a need for further research and debate on the role and value of full autopsies and issues of quality and standards.

## Supporting Information

Table S1 Characteristics of the victim, suspect/perpetrator and case investigation by whether or not the suspect/perpetrator was charged and whether there was a conviction among those charged (weighted data/unadjusted odds ratios).
(DOCX)

Table S2 The distribution of indicators off forensic pathology variables and forensic management of female homicide cases by suspect/perpetrator charged and suspect/perpetrator convicted (weighted data/unadjusted odds ratios).
(DOCX)
