# Supplementary material for: Forensic Medicine in South Africa: Associations between Medical Practice and Legal Case Progression and Outcomes in Female Murders
Source: PLoS One. 2011 Dec 14;6(12):e28620. doi: 10.1371/journal.pone.0028620 (PMC3237462; doi:10.1371/journal.pone.0028620)
Supplement: Table S1 — Characteristics of the victim, suspect/perpetrator and case investigation by whether or not the suspect/perpetrator was charged and whether there was a conviction among those charged (weighted data/unadjusted odds ratios). (DOCX) [file pone.0028620.s001.docx]

**Table S1: Characteristics of the victim, suspect/perpetrator and case investigation by whether or not the suspect/perpetrator was charged and whether there**

**was a conviction among those charged (weighted data/unadjusted odds ratios)**

|  | **Charged** | | | **Convicted** | | |
| --- | --- | --- | --- | --- | --- | --- |
|  | **Charged Yes** | **Charged No** | **Charged** | **Convicted Yes** | **Convicted No** | **Convicted** |
|  |  |  |  |  |  |  |
|  | **% (n=1766)** | **% (n=1156)** | **OR (95%CI)** | **% (n=910)** | **% (n=442)** | **OR (95%CI)** |
| Mean age of victim | 35.7 | 39.9 | 0.98 (0.97-0.99) | 34.8 | 34.9 | 0.99 (0.97-1.01) |
| Mean age of suspect/perpetrator | 32.8 | 35.4 | 0.98 (0.94-1.01) | 32.3 | 35.1 | 0.97(0.94-1.01) |
| **Victim’s race** |  |  |  |  |  |  |
| African | 73.7 | 84.6 | 1 | 66.2 | 84.5 | 1 |
| Coloured | 21.1 | 8.5 | 2.85 (1.52-5.35) | 26.1 | 11.8 | 2.83 (1.12-7.11) |
| White | 4.6 | 5.9 | 0.88 (0.41-1.90) | 7.3 | 2.2 | 4.22 (0.86-20.48) |
| Indian | 0.6 | 1 | 0.73 (0.12-4.22) | 0.4 | 1.5 | 0.33 (0.19-0.58) |
| **Suspect/Perpetrator’s race** |  |  |  |  |  |  |
| African | 76.5 | 86.8 | 1 | 70.9 | 85.5 | 1 |
| Coloured | 19.6 | 10.6 | 2.09 (1.04-4.21) | 25.1 | 8.0 | 3.75 (1.21-11.61) |
| White | 3.1 | 2.5 | 1.36 (0.39-4.68) | 3.4 | 4.9 | 0.82 (0.15-4.36) |
| Indian | 0.7 | 0 | - | 0.6 | 1.6 | 0.47 (0.16-1.39) |
| **Relationship between victim and suspect/perpetrator** |  |  |  |  |  |  |
| Not intimate | 49.0 | 72.8 | 1 | 48 | 48.3 | 1 |
| Intimate | 51.0 | 27.2 | 2.77 (1.40-5.48) | 52 | 51.7 | 1.01 (0.53-1.93) |
| **History of intimate partner violence** |  |  |  |  |  |  |
| No | 81.5 | 94.3 | 1 | 74.6 | 86.8 | 1 |
| Yes | 18.5 | 5.7 | 3.75 (1.48-9.48) | 25.4 | 13.2 | 2.23 (1.01-4.89) |
| **Scene of crime** |  |  |  |  |  |  |
| Not victim’s home | 40.8 | 56.8 | 1 | 42.4 | 34.1 | 1 |
| Victims’ home | 59.2 | 43.2 | 1.90 (1.07-3.40) | 57.6 | 65.9 | 0.70 (0.37-1.33) |
| **Crime scene visited by Investigating Officer** |  |  |  |  |  |  |
| No | 5.8 | 10.1 | 1 | 2.1 | 9.5 | 1 |
| Yes | 94.2 | 89.9 | 1.82 (0.61-5.36) | 97.9 | 90.5 | 5.00 (1.78-14.04) |
| **Crime scene photos taken** |  |  |  |  |  |  |
| No | 31.2 | 36 | 1 | 25.2 | 30.8 | 1 |
| Yes | 68.8 | 64 | 1.24 (0.68-2.24) | 74.8 | 69.2 | 1.31 (0.48-3.55) |
| **Weapon found** |  |  |  |  |  |  |
| No | 48. 3 | 93.7 | 1 | 34.9 | 61.4 | 1 |
| Yes | 51.7 | 6.3 | 16.02 (8.66-29.64) | 65.1 | 38.6 | 2.97 (1.46-6.01) |
