# Supplementary material for: Forensic Medicine in South Africa: Associations between Medical Practice and Legal Case Progression and Outcomes in Female Murders
Source: PLoS One. 2011 Dec 14;6(12):e28620. doi: 10.1371/journal.pone.0028620 (PMC3237462; doi:10.1371/journal.pone.0028620)
Supplement: Table S2 — The distribution of indicators off forensic pathology variables and forensic management of female homicide cases by suspect/perpetrator charged and suspect/perpetrator convicted (weighted data/unadjusted odds ratios). (DOCX) [file pone.0028620.s002.docx]

**Table S2: The distribution of indicators off forensic pathology variables and forensic management of female homicide cases by suspect/perpetrator charged and suspect/perpetrator convicted (weighted data/unadjusted odds ratios )**

|  | Charged Yes | Charged No | Charged | Convicted Yes | Convicted No | Convicted |
| --- | --- | --- | --- | --- | --- | --- |
|  | % (n=1770) | % (n=1150) | OR (95%CI) | % (n=910) | % (n=442) | OR (95%CI) |
| **Full autopsy done** |  |  |  |  |  |  |
| **No** | 30.3 | 27.6 | 1 | 33.6 | 24.5 | 1 |
| **Yes** | 69.7 | 72.4 | 0.87 (0.45-1.68) | 66.4 | 75.5 | 0.64 (0.17-2.36) |
| **Autopsy done at academic centre** |  |  |  |  |  |  |
| **No** | 81.0 | 76.4 | 1 | 80.2 | 83.7 | 1 |
| **Yes** | 19.0 | 23.4 | 0.76 (0.33-1.78) | 19.8 | 16.3 | 1.27 (0.65-2.46) |
| **Qualification of Medical practitioner** |  |  |  |  |  |  |
| **Specialist** | 19.8 | 17.1 | 1 | 18.1 | 20.2 | 1 |
| **Some training** | 14.5 | 16.4 | 0.76 (0.43-1.33) | 14.7 | 10.1 | 1.63 (0.62-4.27) |
| **No training** | 65.6 | 66.5 | 0.84 (0.42-1.67) | 67.2 | 69.7 | 1.07 (0.46-2.47) |
| **Crime scene visited by medical practitioner** |  |  |  |  |  |  |
| **No** | 99.0 | 99.4 | 1 | 99.4 | 98.8 | 1 |
| **Yes** | 1.0 | 0.6 | 1.46 (0.68-3.11) | 0.6 | 1.2 | 0.53 (0.08-3.27) |
| **Forensic photos taken during autopsy** |  |  |  |  |  |  |
| **No** | 84.3 | 89.2 | 1 | 84.4 | 88.4 | 1 |
| **Yes** | 15.7 | 10.8) | 1. 53(0.74-3.18) | 15.6 | 11.6 | 1.41 (0.81-2.44) |
| **Specimen collected for evidence** |  |  |  |  |  |  |
| **Specimen collected that could be used for DNA analysis** | 11.8 | 23.2 | 0.44 (0.26-0.72) | 13.4 | 6.2 | 2.34 (0.98-5.54) |
| **Histology specimen collected** | 1.3 | 1.4 | 0.91 (0.57-1.44) | 0.9 | 1.9 | 0.44 (0.15-1.21) |
| **Toxicology specimen collected** | 0.4 | 2.4 | 0.17 (0.02-1.13) | 0.2 | 1.2 | 0.18 (0.02-1.65) |
| **Genital swab specimen collected** | 9.5 | 20.2 | 0.41 (0.22-0.76) | 11 | 5.4 | 2.14 (0.80-5.74) |
| **Clothes specimen collected** | 2.5 | 1.9 | 1.31 (0.71-2.44) | 1.8 | 1.5 | 1.20 (0.26-5.48) |
| **Head hair specimen collected** | 4.4 | 7.2 | 0.58 (0.33-1.02) | 6.1 | 2.3 | 2.74 (0.85-8.80) |
| **Nail scrapings specimen collected** | 4.9 | 6.7 | 0.71 (0.35-1.44) | 6.3 | 1.5 | 4.30 (1.36-13.53) |
| **Victim blood alcohol collected** | 44.6 | 34.1 | 1.55 (0.81-2.97) | 47.5 | 39.8 | 1.36 (0.63-2.92) |
| **Evidence of rape reported** |  |  |  |  |  |  |
| **No** | 88.7 | 76.0 | 1 | 84.8 | 93.0 | 1 |
| **Yes** | 11.3 | 24.0 | 0.40 (0.18-0.87) | 15.2 | 7.0 | 2.40 (0.96-5.96) |
| **Multiple injuries** |  |  |  |  |  |  |
| **No** | 57.5 | 56.6 | 1 | 55.8 | 53.6 | 1 |
| **Yes** | 42.5 | 43.4 | 0.96 (0.60-1.52) | 44.2 | 46.4 | 0.91 (0.44-1.85) |
| **Standard of post mortem report** |  |  |  |  |  |  |
| **Poor** | 39.3 | 39.1 | 1 | 46.1 | 33.1 | 1 |
| **Adequate** | 32.1 | 34.1 | 0.93(0.53-1.10) | 25.7 | 38.5 | 0.67 (0.34-1.31) |
| **Superior** | 28.6 | 26.8 | 1.05 (0.53-1.61) | 28.2 | 28.4 | 1.40 (0.40-4.90) |
